# Phase II study of everolimus (RAD001) monotherapy as first-line treatment in advanced biliary tract cancer with biomarker exploration: the RADiChol Study

**DOI:** 10.1038/s41416-018-0021-1

**Published:** 2018-03-12

**Authors:** David K. Lau, Rebecca Y. Tay, Yvonne H. Yeung, Fiona Chionh, Jennifer Mooi, Carmel Murone, Effie Skrinos, Timothy J. Price, John M. Mariadason, Niall C. Tebbutt

**Affiliations:** 1 Olivia Newton-John Cancer Research Institute, Melbourne, VIC Australia; 2 School of Cancer Medicine, La Trobe University, Bundoora, VIC Australia; 3 Dept of Medical Oncology, Olivia Newton-John Cancer Wellness and Research Centre, Austin Health, Melbourne, VIC Australia; 4 The Queen Elizabeth Hospital and University of Adelaide, Woodville, SA Australia

**Keywords:** Targeted therapies, Prognostic markers

## Abstract

**Background:**

Advanced biliary tract cancers (BTCs) have a poor prognosis and limited treatment options. This exploratory phase II study aimed to evaluate the activity of the mTOR inhibitor everolimus in advanced BTC and explore prognostic biomarkers.

**Methods:**

Patients with advanced BTCs, who had not received chemotherapy for advanced disease, were enroled to receive everolimus (10 mg daily). The primary endpoint was disease control rate (DCR) at 12 weeks. Secondary endpoints included overall response rate, progression-free survival (PFS), overall survival (OS) and adverse events. Activation status of the RAS and phosphatidylinositol 3-kinase (PI3K)/AKT/mTOR pathways was assessed by DNA sequencing and immunohistochemistry on archival tumour tissue.

**Results:**

The study enroled 27 patients and the DCR at 12 weeks was 48%. Median PFS was 5.5 months (95% confidence interval (CI): 2.1–10.0 months) and median OS was 9.5 months (95% CI: 5.5–16.6 months). DCR at 12 weeks was significantly worse for gall bladder carcinoma compared to other anatomical sites, and there was a trend towards a worsened PFS and OS. Treatment was well tolerated. *KRAS* (12%) and *PIK3CA* mutations (12%) were uncommon. Immunohistochemical staining for PI3K/AKT/mTOR pathways did not significantly correlate with outcome.

**Conclusion:**

In unselected patients, everolimus demonstrated clinical activity as first-line monotherapy in advanced BTC.

## Introduction

Biliary tract cancers (BTCs) are a heterogenous group of tumours of the intrahepatic and extrahepatic biliary system and the gall bladder. Advanced or unresectable locally advanced disease has a poor prognosis with limited systemic treatment options.^1^ Combination platinum-gemcitabine chemotherapy is an active first-line treatment regimen;^2^ however, it is associated with low rates of radiologic response and short time to tumour progression. Effective new treatments are therefore urgently required.

Everolimus is a derivative of rapamycin that selectively inhibits mTORC1 (mammalian target of rapamycin complex 1), a key protein kinase complex which regulates cell growth, proliferation and survival. Activation of mTORC1 is mediated by the phosphatidylinositol 3-kinase (PI3K) pathway through activation of AKT/PKB and subsequent inhibition of the tuberous sclerosis complex 1/2 (TSC1/2).^3^ S6 kinase and 4EBP1 are key downstream effectors of this pathway and are critical regulators of protein synthesis.

Constitutive activation of the PI3K/AKT/mTOR pathway due to genetic alterations in *PIK3CA*,^4,5^ *PTEN*,^6^ *PIK3R1*,^5^ *PI3KC2G*,^7^ *AKT1*,^6^ *AKT3*^5^ and *TSC1*^5^ collectively occurs in ~10–20% BTCs. In addition, immunohistochemical (IHC) staining of PI3K pathway components phosphorylated (p)-AKT and p-mTOR suggests that up to 50% of BTCs may harbour activation of this pathway,^8,9^ which is associated with worse survival outcomes.^10–12^ Finally, constitutive activation of the PI3K pathway contributes to the development of BTC in genetic mouse models,^13^ making mTOR inhibition an attractive therapeutic target in BTC.

In this regard, we and others have recently demonstrated that mTOR inhibitors, including everolimus, inhibit the proliferation of BTC cell lines in vitro and in vivo.^9,14–17^ Biomarker studies in a panel of BTC cell lines revealed mutations and/or amplification of *KRAS* to be associated with resistance, and high basal p-AKT levels to be associated with response to everolimus.^17^ Activating *KRAS* mutations are present in 16–22% of BTCs.^18–20^

mTOR inhibitors are approved for the treatment of renal cell, neuroendocrine and hormone-positive/HER-2-negative advanced breast cancer.^21–23^ Recently, the phase II Italian Trials in Medical Oncology (ITMO) trial assessed everolimus activity in patients with chemorefractory advanced BTC, where a favourable toxicity profile and encouraging anti-tumour activity was reported.^24^

The objective of this exploratory single-arm phase II study, therefore, was to evaluate the clinical activity and safety of everolimus in advanced cholangiocarcinoma in the first-line setting. In addition, we sought to identify potential prognostic and predictive biomarkers of response.

## Materials and methods

### Eligibility

This study enroled patients of any age with metastatic or locally advanced (unresectable) adenocarcinoma of the gall bladder, intrahepatic or extrahepatic biliary tract, or ampulla of Vater, as confirmed by histology or cytology. Eligible patients had an Eastern Cooperative Oncology Group (ECOG) performance status of 0, 1 or 2; no prior chemotherapy for advanced BTC (prior adjuvant chemotherapy allowed), and adequate organ function (defined as creatinine ≤1.5 × ULN (upper limit of normal), bilirubin ≤1.5 x ULN, neutrophils ≥1.5, platelets ≥100). Measurable or non-measurable disease were allowed.

Patients were excluded if they had received any prior mTOR inhibitor therapy or had intolerance or hypersensitivity to rapalogs; severe and/or uncontrolled diabetes mellitus, hyperlipidemia, liver disease, infection or impaired pulmonary function and/or prior history of another malignancy (with the exception of non-melanoma skin cancer, carcinoma in situ of the uterine cervix or any other cancer treated with curative intent without evidence of relapse for more than 2 years).

### Study protocol

All patients received oral everolimus at a starting dose of 10 mg per day until tumour progression, unacceptable toxicity or at the discretion of the investigator.

Clinical review and assessment for toxicity was undertaken every 3 weeks. Fasting glucose and lipid profile and computerised tomography (CT) was performed every 6 weeks. Tumour response was assessed according to Response Evaluation Criteria in Solid Tumours (RECIST) 1.0 criteria.

Two dose reductions, 5 mg daily, then 5 mg/s daily, were allowed in the event of drug toxicity or treatment interruption. Patients requiring a third dose reduction were required to discontinue study treatment.

The primary endpoint was disease control rate (DCR) at 12 weeks, which was defined as the proportion of patients with complete response, partial response or stable disease in the intention-to-treat population. Secondary endpoints included objective response rate (ORR), progression-free survival (PFS), overall survival (OS) and grade 3 or 4 adverse events according to NCI Common Terminology Criteria for Adverse Event v3.0. PFS was defined as the time to disease progression, the occurrence of new disease or death from any cause. Exploratory endpoints included evaluation of intra-tumoural expression of components of the PI3K/AKT/mTOR signalling pathway, *KRAS* and *PIK3CA* mutation status and *KRAS* amplification status, and correlation with DCR, PFS and OS.

Novartis provided the study drug and an unrestricted grant but was not involved in the analysis or interpretation of the data or the writing of the manuscript. The RADiChol study (ClinicalTrials.gov identifier: NCT00973713) was performed in accordance with the NHMRC Statement on Ethical Conduct in Research Involving Humans 1999 (© Commonwealth of Australia 1999) and the principles laid down by the World Medical Assembly in the Declaration of Helsinki and complied with ICH GCP Guidelines. The protocol was approved by the Human Research and Ethics Committee at both study sites. Patients provided written informed consent for study participation and donation of tumour tissue for exploratory biomarker analysis prior to enrolment.

### Biomarker analysis

Formalin-fixed, paraffin-embedded (FFPE) archival tumour tissue was available from 21 of 27 study participants for IHC staining analyses. Briefly, 4 μm sections were cut onto charged glass slides. Slides underwent microwave heat-induced epitope retrieval in sodium citrate buffer (pH 6.0) or Tris-EDTA (pH 9.0), followed by incubation with primary antibodies. Sections were incubated with Real Envision-HRP secondary antibody, diaminobenzidine substrate (DAKO) and counterstained with haematoxylin. Positive and negative controls from a BTC cell line microarray were used for each antibody to validate the staining protocol. Scoring was based on the strongest cytoplasmic staining intensity in each tumour section, scored as 0 (absent), 1+ (weak), 2+ (moderate) or 3+ (strong). Scoring was performed by two investigators who were blinded to the clinical outcomes. For clinical correlation, expression was categorised into low (0 or 1+) and high expression (2+ or 3+). Primary antibodies against phospho-S6 Ser 235/236 (cat. no. 4858), phospho-S6 Ser 240/244 (5364), phospho-mTOR Ser 2448 (2976), phospho-AKT Ser 473 (4060) and phospho-4EBP1 T37/46 (2855) were obtained from Cell Signalling Technology (Beverly, MA, USA). Anti-PTEN (6H2.1) was purchased from Cascade Bioscience (Winchester, MA, USA).

### Mutation analysis

Genomic DNA was macrodissected from FFPE tissue blocked and extracted using the QIAmp DNA FFPE Tissue Kit (Qiagen, Germany). KRAS and PIK3CA genotyping were determined by amplification of KRAS and PIK3CA using Platinum Taq DNA Polymerase High Fidelity (Invitrogen) and conventional Sanger sequencing at the Australian Genomic Research Facility. The primer sequences were as follows: KRAS exon 2F: 5′-AGTCACATTTTCATTATTTT-3′, R: 5′-AGAAACCTTTATCTGTATCAAAGAATG-3′; KRAS exon 3F: 5′-CACTGTAATAATCCAGACTGTG-3′, R: 5′-CTATAATTACTCCTTAATGTCAGC-3′; PIK3CA exon 9F: 5′-TGTGAATCCAGAGGGGAAAA-3′, R: 5′-AAATTCAGTTATTTTTTCTG-3′; exon 20F: 5′-GCTCCAAACTGACCAAACTG-3′, R: 5′-CTGTTTAATTGTGTGGAAGA-3′.

### Fluorescent in situ hybridisation

Fluorescent dual probes for KRAS and CEN12 were applied to FFPE tissue with ZytoLight Fluorescent in situ hybridisation (FISH)-Tissue Implementation Kit (Zytovision, Germany) as per the manufacturer’s instructions and visualised with a Carl-Zeiss AXISKOP2 Microscope.

### Statistical analysis

Based on pre-clinical findings, it was anticipated that cytostatic treatments such as mTOR inhibitors would have the greatest impact on disease control (i.e., inhibition of tumour progression). Therefore, the primary endpoint was DCR at 12 weeks. It was assumed that a DCR of 60% at 12 weeks would be of interest and that a DCR of 35% would be of little interest. The sample size was based upon a Simon’s two-stage design to reject the null hypothesis with 80% power. The study would proceed past the first stage if >3 out of 9 patients had disease control at 12 weeks, and would be declared active if >13 out of 27 patients had disease control at 12 weeks.

The final efficacy analysis was by intention to treat. PFS and OS were analysed by the Kaplan–Meier method. DCR confidence intervals were determined by the Clopper–Pearson method. Toxicity was analysed by treatment received. Fisher’s exact test was used to determine the relationship between specific biomarkers and DCR, and a log-rank test was used to determine the relationship with PFS and OS. All *P* values were two-sided and a *P* value <0.05 was considered statistically significant. Statistical analysis was performed on R-studio version 0.99.447.

## Results

### Patient characteristics

A total of 27 patients were enroled between September 2009 and December 2011 at two institutions—Austin Health, Melbourne (*n* = 26) and Queen Elizabeth Hospital, Adelaide (*n* = 1), and all patients received at least one dose of everolimus at a starting dose of 10 mg per day.

Baseline patient characteristics are outlined in Table 1. The median age was 64 years, 41% of patients were male (*n* = 11) and 93% had an ECOG performance status of 0–1. Two patients had received adjuvant chemotherapy with single-agent gemcitabine or fluorouracil.

**Table 1:** Baseline characteristics of study participants

	*n* (%)
Number of patients	27
Age, years (range)	64 (34–84)
Male	11 (41%)
Performance status (ECOG)	
0	9 (33%)
1	16 (59%)
2	2 (7%)
Tumour location	
Intrahepatic	6 (22%)
Extrahepatic/hilar	6 (22%)
Gall bladder	12 (44%)
Ampulla of Vater	2 (7%)
Unknown	1 (4%)
Prior therapies	
Curative surgery	4 (15%)
Palliative surgery	6 (22%)
Adjuvant chemotherapy	2 (7%)
Biliary stent	8 (30%)
Radiotherapy	0 (0%)

*ECOG* Eastern Cooperative Oncology Group

The median time on study was 3.8 months (range 0.6–21.0). Nineteen patients discontinued the study drug due to tumour progression. Four patients ceased treatment due to toxicity, including one patient within 12 weeks of enrolment. Two patients withdrew consent and two patients died due to tumour progression on study.

### Efficacy analysis

The first part of the study was passed with 3/9 patients demonstrating disease control at 12 weeks. The study therefore proceeded to the second stage, enroling a total of 27 patients.

**Fig. 1:**
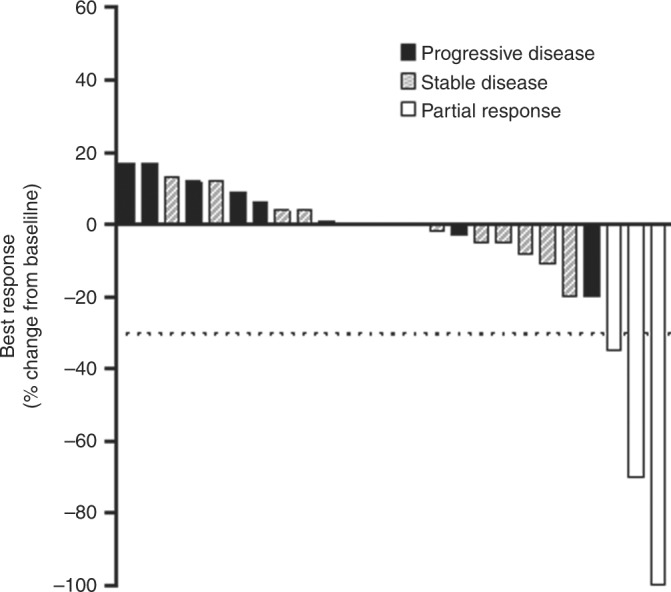
Objective tumour responses with everolimus. Waterfall plot of the maximum percentage change in target lesions compared with baseline measurements. Three patients achieved a partial response. Four patients had a 0% change in tumour burden. The dotted line denotes a 30% reduction in target lesions

The primary endpoint, DCR at 12 weeks was 48% (*n* = 13/27, 95% CI: 28.7–68.0). Twenty-five patients had evaluable disease by RECIST 1.0. There were three partial responses and no complete responses (ORR 12%, 95% CI: 3–31%). There were 15 (60%) patients who achieved stable disease; however, five of these patients had clinically progressed at 12 weeks. Seven patients (28%) had progressive disease (Fig. 1).

**Fig. 2:**
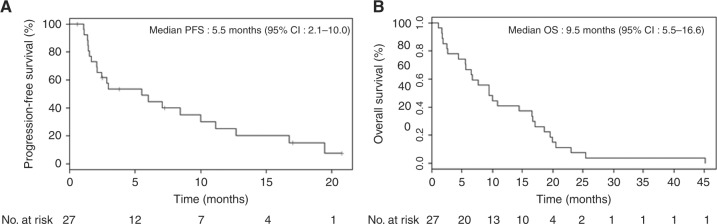
Survival outcomes in patients with advanced biliary tract cancer who received everolimus. Kaplan–Meier estimates of progression-free survival (**a**) and overall survival (**b**). CI confidence interval

Median PFS was 5.5 months (95% CI: 2.0–10.0 months) and median OS was 9.5 months (95% CI: 5.5–16.6 months) (Fig. 2). On progression, 10 patients (37%) proceeded to second-line therapy with cytotoxic chemotherapy.

DCR at 12 weeks for gall bladder carcinoma was 25% versus non-gall bladder 71% (*P* = 0.047). Median PFS for gall bladder carcinoma was 2.1 months (95% CI: 1.4–6.0) and for non-gall bladder carcinoma 8.4 months (95% CI: 1.4–12.7) but was not significantly different (*P = *0.395). Median OS for gall bladder and non-gall bladder carcinoma was 5.6 months (95% CI: 1.8–17.1) and 15.5 months (95% CI: 6.7–19.9) respectively (*P* = 0.092).

### Adverse events

The most common drug-related toxicity was oral mucositis/stomatitis (63%). Expected drug-related toxicities of any grade were rash (52%), thrombocytopenia (7%), neutropenia (7%), anaemia (19%), hyperglycaemia (22%), hypercholesterolaemia (19%) and pneumonitis (15%) (Table 2).

**Table 2:** Adverse events according to Common Terminology Criteria for Adverse Events (CTCAE) v3.0

	All grades	Grade 3 or 4
Adverse event	*n* (%)	*n* (%)
Stomatitis/oral mucositis	17 (63%)	2 (7%)
Pain	16 (59%)	4 (15%)
Rash	14 (52%)	1 (4%)
Nausea	12 (44%)	0 (0%)
Diarrhoea	12 (44%)	1 (4%)
Infections	11 (41%)	7 (26%)
Fatigue	10 (37%)	1 (4%)
Cough	9 (33%)	0 (0%)
Anorexia	8 (30%)	1 (4%)
Vomiting	8 (30%)	0 (0%)
Pruritus	7 (26%)	0 (0%)
Oedema	7 (26%)	0 (0%)
Hyperglycaemia	6 (22%)	3 (11%)
Weight loss	6 (22%)	0 (0%)
Epistaxis	6 (22%)	0 (0%)
Anaemia	5 (19%)	3 (11%)
Hypercholesterolaemia	5 (19%)	0 (0%)
Hypokalaemia	5 (19%)	1 (4%)
Taste alteration	4 (15%)	0 (0%)
Pneumonitis	4 (15%)	1 (4%)
Dry skin	4 (15%)	0 (0%)
Nail changes	4 (15%)	0 (0%)
Insomnia	4 (15%)	0 (0%)
Constipation	3 (11%)	1 (4%)
Headache	3 (11%)	0 (0%)
Neutropenia	2 (7%)	0 (0%)
Thrombocytopenia	2 (7%)	1 (4%)
Flu-like syndrome	2 (7%)	0 (0%)
Thromboembolism	2 (7%)	2 (7%)
Ocular toxicity	2 (7%)	0 (0%)

The most frequently reported grade 3/4 adverse events were infection (26%), pain (15%), hyperglycaemia (11%) and anaemia (11%). Of the 70.4% of patients with any grade 3/4 adverse events, most were attributed to underlying disease, not drug toxicity. No unexpected treatment-related toxicities or deaths were reported.

### Biomarker analyses

#### Analysis of the PI3K/AKT/mTOR pathway

As everolimus targets the PI3K/AKT/mTOR pathway, we performed an exploratory biomarker analysis to determine whether the activation status of the pathway correlated with drug efficacy. Tumour tissue was available from 21 of the 27 participants (78%) for biomarker analysis by immunohistochemistry, and sufficient DNA for mutation status analysis was obtained from 16 patients.

Two patients harbouring activating hotspot mutations in *PIK3CA* were identified. One of these patients who had a previously resected T2N0 ampullary carcinoma with intestinal differentiation and received adjuvant gemcitabine 2 years before enrolment in this study had a prolonged PFS on everolimus (16.7 months).

**Fig. 3:**
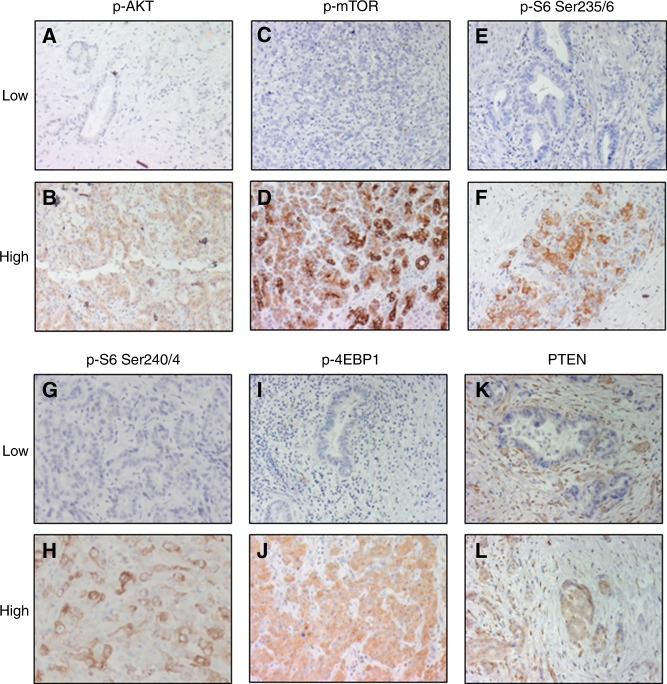
Immunohistochemistry staining of biliary tract cancer tissue from the RADICHOL trial. Representative low and high expression of p-AKT (**a**,** b**), p-mTOR (**c**, **d**), p-S6 Ser 235/236 (**e**, **f**), p-S6 Ser 240/244 (**g**, **h**), p-4EBP1 (**i**, **j**) and PTEN (**k**, **l**); 10× magnification

Activation status of the PI3K/AKT/mTOR pathway was also determined immunohistochemically by staining for basal expression of the target of rapamycin, p-mTOR, the upstream pathway components PTEN and p-AKT (Ser 473) and the downstream components p-S6 (Ser 235/236), p-S6 (240/244) and p-4EBP1 (T37/46) (Fig. 3)

We first assessed whether the pathway is activated in a linear manner in BTCs by examining the correlation between markers. While overexpression of p-S6 (Ser 235/236) was elevated in the two patients harbouring *PIK3CA* mutations, staining intensity of p-AKT, p-mTOR, p-S6 (Ser 235/236), p-S6 (Ser 240/244) and p-4EBP1 did not correlate across the tumours, indicating a complex mode of pathway activation.

Assessment of the association between marker expression and everolimus response identified a trend towards worse OS in patients with high tumour p-4EBP1 staining (*P* = 0.052) (Table 3). There was no statistically significant correlation between staining intensity of p-AKT, p-mTOR, p-S6 (Ser 235/236) or p-S6 (Ser 240/244) and efficacy outcomes.

**Table 3:** Association of markers of PI3K/AKT/mTOR pathway activity with clinical outcome

Basal biomarker	*n* (%)	DCR at 12 weeks (%)	*P* value	Median PFS (95% CI), months	*P* value	Median OS (95% CI), months	*P* value
p-S6 Ser 235/236							
Low	14 (67)	6 (43)	0.659	3.0 (1.4–12.7)	0.792	8.5 (2.5–16.6)	0.281
High	7 (33)	4 (57)		6.0 (1.1–NR)		9.5 (1.1–20.5)	
p-S6 Ser 240/244							
Low	13 (62)	7 (54)	0.659	5.8 (1.4–12.7)	0.471	14.4 (2.47–18.6)	0.378
High	8 (38)	3 (37)		3.0 (1.1–NR)		6.6 (1.09–16.6)	
p-mTOR							
Low	15 (68)	6 (40)	0.685	5.5 (1.4–12.7)	0.295	9.5 (5.5–16.6)	0.851
High	7 (32)	3 (43)		2.5 (1.1–8.4)		6.7 (1.1–17.1)	
p-AKT Ser 473							
Low	14 (67)	7 (50)	1	5.5 (1.4–11.2)	0.409	8.1 (1.81–16.6)	0.853
High	7 (33)	3 (43)		6.0 (1.41–NR)		9.5 (4.38–19.6)	
p-4EBP1							
Low	13 (62)	8 (61)	0.183	6.0 (1.4–16.8)	0.143	14.4 (2.5–19.9)	0.052
High	8 (38)	2 (25)		2.6 (1.09–NR)		6.6 (1.09–16.5)	
PTEN							
Low	5 (24)	3 (60)	0.635	7.1 (2.8–NR)	0.844	16.6 (5.6–NR)	0.621
High	16 (76)	7 (44)		5.5 (1.4–16.8)		7.2 (2.5–17.1)	

*CI* confidence interval, *NR* not reached

#### Analysis of *KRAS* amplification and mutation status

Mutations in *KRAS* have been associated with resistance to everolimus in BTC cell lines,^17^ and in patients with colorectal cancer who are treated with this agent.^25^ Sanger sequencing of *KRAS* (exons 2 and 3) identified only two patients harbouring *KRAS* mutations, of whom one was the patient with ampullary carcinoma with intestinal differentiation who also harboured a *PIK3CA* (E545K) mutation. *KRAS* amplification has previously been reported in a small subset of BTC patients;^20^ however, no patients with *KRAS* amplification using FISH were identified in the current study. Collectively, the low prevalence of *KRAS* mutations and amplification precluded investigation of associations with outcome.

## Discussion

While this study narrowly failed to meet its primary endpoint of >13/27 patients experiencing disease control at 12 weeks, everolimus monotherapy did have clinical activity in a subset of patients with advanced BTC in the first-line setting.

The progression-free and OS outcomes observed in this study (5.5 and 9.5 months, respectively) were comparable to results from the ITMO phase II single-arm trial of everolimus monotherapy in BTC following progression on chemotherapy, which reported an 8-week DCR of 44.4%, PFS of 3.2 months, and OS 7.7 months.^24^ Nevertheless, platinum-gemcitabine doublet chemotherapy remains the most active regimen in an unselected population of advanced BTC (median PFS 8.0 months, median OS 11.7 months).^2^

DCR at 12 weeks was significantly worse for gall bladder carcinoma, and there was a trend towards a worsened PFS and OS. When compared to other anatomical sites, gall bladder carcinoma is not regarded as an adverse prognostic marker in BTC,^26,27^ suggesting that gall bladder carcinoma may be resistant to everolimus. While our study lacked statistical power, this result warrants further investigation.

The adverse event profile for everolimus was also similar to that previously reported in other studies.^21–23^ Oral mucositis/stomatitis was the most common drug-related toxicity (63%), which was most frequently grades 1 and 2. Conservative measures with a non-alcoholic mouthwash or topical analgesic mouth treatment can successfully manage this oral toxicity. One event of G3 pneumonitis was reported, necessitating treatment interruption. The favourable overall toxicity profile of everolimus suggests that it can be safely administered in the outpatient setting for advanced BTC.

An important feature of this study was an exploratory analysis of potential biomarkers of everolimus response. In this regard, we recently observed that BTC cell lines with high basal p-AKT expression respond preferentially to everolimus in vitro.^28^ Similarly, renal and gastric cancers harbouring higher basal activation of the PI3K/AKT/mTOR pathway have also been reported to show improved response to everolimus.^29,30^

In comparison, in the current study we failed to observe an association between the levels of a number of IHC readouts of PI3K/AKT/mTOR pathway activity and benefit to everolimus treatment. One limitation of this approach was the lack of concordance between the different markers of PI3K/AKT/mTOR pathway activity. This may reflect the complex biological regulation of this pathway in BTC, such as possible activation by multiple upstream effectors and crosstalk with other pathways including mitogen-activated protein kinase. Continued investigation of more robust biomarkers of everolimus is therefore required. However, we also acknowledge that the study lacked sufficient statistical power to definitively determine the predictive power of these markers and their potential to predict everolimus sensitivity should not be excluded.

Nevertheless, the biomarker analysis did reveal two potentially interesting findings. First, although the relatively low frequency of *KRAS* and *PIK3CA* mutations in this cohort (12.5%) precluded assessment of an association with outcome, we noted that one patient with ampullary carcinoma with intestinal differentiation who had a concurrent *PIK3CA* and *KRAS* mutation had a prolonged PFS of 16.7 months. In comparison to other BTCs, ampullary carcinomas have a higher rate of *PIK3CA* (13%) and *KRAS* (54%) mutations, and these mutations have been reported to co-exist.^31^ Whether the response of this patient to everolimus is a consequence of their *PIK3CA* mutation status, or reflective of other biological features unique to ampullary carcinoma requires additional investigation.

Second, we observed that high tumour expression of p-4EBP1 was associated with a worse outcome. However, the lack of a control arm on the current study does not allow for distinction between whether high p-4EBP is predictive of resistance to everolimus or prognostic of poor outcome. In this regard, it is notable that several previous studies have linked hyperactivation of the PI3K/AKT/mTOR pathway as assessed by IHC measurement of p-mTOR, PTEN and p-4EBP1 with a poorer prognosis in BTC.^10–12^

In this exploratory phase II trial, although single-agent everolimus narrowly failed to meet its primary endpoint, favourable anti-tumour activity was observed in a subset of patients with advanced BTCs. Further studies of everolimus in BTC are warranted with ongoing focus on identifying predictive biomarkers.
